# Onshore industrial wind turbine locations for the United States up to March 2014

**DOI:** 10.1038/sdata.2015.60

**Published:** 2015-11-24

**Authors:** Jay E. Diffendorfer, Louisa A. Kramer, Zach H. Ancona, Christopher P. Garrity

**Affiliations:** 1 US Geological Survey, Geosciences and Environmental Change Science Center, Denver, CO, 80225, USA; 2 US Geological Survey, Eastern Energy Resources Science Center, Reston, VA, 20192, USA

**Keywords:** Atmospheric dynamics, Technology, Conservation biology, Geography, Environmental sciences

## Abstract

Wind energy is a rapidly growing form of renewable energy in the United States. While summary information on the total amounts of installed capacity are available by state, a free, centralized, national, turbine-level, geospatial dataset useful for scientific research, land and resource management, and other uses did not exist. Available in multiple formats and in a web application, these public domain data provide industrial-scale onshore wind turbine locations in the United States up to March 2014, corresponding facility information, and turbine technical specifications. Wind turbine records have been collected and compiled from various public sources, digitized or position verified from aerial imagery, and quality assured and quality controlled. Technical specifications for turbines were assigned based on the wind turbine make and model as described in public literature. In some cases, turbines were not seen in imagery or turbine information did not exist or was difficult to obtain. Uncertainty associated with these is recorded in a confidence rating.

## Background & Summary

Wind-powered electricity generation has increased to an installed cumulative capacity of 61.5 gigawatts (GW) by September, 2014 (ref. 1), accounting for 31% of the US electricity production from renewable sources. Wind energy is currently the second largest form of renewable electric generation, behind hydroelectric generation, with installed capacity growing faster than other types of renewable energy^2^.

The growth of wind energy as a sustainable energy source has led to scientific and engineering questions that require detailed spatial information on turbines. For example, a number of scientists study the wakes generated by wind turbines and how these alter microclimates^3,4^. In addition, turbine placement within the electricity transmission grid requires technological solutions to balance loads^5^ and can result in interactions between wind farms through wake externalities^6^.

Regulators are also involved with the placement of wind turbines in sensitive locations. The Department of Defense is concerned about wind turbine effects on radar and low-level flight training areas^7^ while the Federal Aviation Administration (FAA) considers wind turbines greater than 200 feet flight obstacles regulated under Title 14 of the Code of Federal Regulations. State and federal agencies as well as conservation organizations are concerned about turbine impacts on species^8,9^. Finally, many county and city planners recognize issues such as noise and visual impacts of wind turbines, which results in a myriad of regulatory and sometimes legal actions^10^. All of these topics require high quality, geospatially accurate locational data on turbines.

The ‘industrial’ turbines mapped are typically large turbines, at least 30.5 m tall, associated with centralized energy-generating facilities. We did not attempt to map small, decentralized turbines, associated with homes, ranches, or other applications. In 2008, U.S. Geological Survey (USGS) scientists began studying energy infrastructure and found sparse and often spatially inaccurate publicly available information regarding wind turbines and their locations. This eventually led to an effort to map wind turbines across the entire US.

The wind turbine dataset includes 48,976 records of individual turbines including their location verified by high-resolution imagery. Additional data includes facility-level information associated with the turbine, turbine specifications, and the confidence in the information provided. This dataset contains records of turbines up to March 2014.

## Methods

Developing the turbine dataset involved starting with existing data on turbine locations gathered from the Federal Aviation Administration (FAA), adding additional turbines based on visual inspections of wind facilities, and three additional processing steps. First, we verified the location of all turbines. Second, we attributed groups of turbines to particular wind facilities. Third, we developed specifications (height, blade length, make, model, etc., see Data Records below) for each turbine. The order of steps was by design. By having verified locations, we could more readily assign turbines to facilities. Once we had turbines linked to a facility, we could use the facility name to search for information about the makes and models of the turbines installed, then use this information to develop the turbine specification.

At the start of the project, 21,419 turbine locations had been digitized using United Stated Department of Agriculture (USDA)/National Resources Conservation Service (NRCS)/National Agriculture Imagery Program (NAIP) orthoimagery during and after an earlier wind facility land transformation study^11^. During this work, a shapefile of turbine locations was created in ArcGIS 10 (ref. 12) by digitizing the base of an observed turbine in the orthoimagery as a point. The shapefile included the turbine location and the facility name but no other information. These turbines were found by visually searching the imagery.

The main source of turbine location data used in the USGS turbine dataset is generated by the Terrain and Obstacles Data (TOD) Team at the FAA. This group maintains a Digital Obstacles File (DOF) dataset of built structures greater than 200 feet and lower structures in the vicinity of some airports that could affect air navigation. The DOF text files, available to the public, are updated both daily and every 56 days and include a field for obstacle type, which contains wind turbines coded as ‘WINDMILL’. Because industry-scale wind turbines are all larger than 200 feet, energy companies report these to the FAA. We filtered the wind turbines in the DOF data and converted the turbine coordinates from degrees, minutes, and seconds to decimal degrees using: decimal degrees=degrees+minutes/60+seconds/3600. In addition, we kept 3 fields from the original DOF: FAA_jdate, FAA_AGL, FAA_ORS (see Data Records, for descriptions). The final text file was converted to a shapefile in ArcGIS 10.

The wind turbine points from the land transformation study were merged with the newly created DOF shapefile. When turbine points in the land transformation shapefile were within 50 m of DOF points (duplicate locations), the verified locations from the land transformation data were combined with the attribute from the DOF. The remaining, non-duplicate turbine locations from the land transformation study (78) were also kept. Turbine points were selected by state, saved as an individual turbine shapefile for each state, and distributed to USGS authors for editing.

Supplemental layers were added to the state-level shapefiles to assist turbine attribution, data validation, and image interpretation. Wind facility data were acquired from the Energy Information Administration’s (EIA) EIA-860 dataset which provides information on existing and planned electricity generating facilities in the US and the now defunct Wind Energy Data and Information (WENDI) Gateway at Oak Ridge National Laboratory. WENDI developed a wind facilities database with included information on the facility name, number of turbines, and turbine manufacturer. These data are now available through OpenEI.org (http://en.openei.org/wiki/Map_of_Wind_Farms). Additional layers included state and county boundaries, as well as cultural, landform, hydrography, and city names from the Homeland Security Infrastructure Program (HSIP) database. HSIP data were used internally to assist with interim steps and are not part of the final product.

Turbine points were visually verified and moved to the base of the turbine as needed. To do so, the turbine point was compared to high-resolution aerial imagery from a variety of sources. No single source was used, because it was not always available at any given place or year. A base map image of Bing Maps Aerial, or other aerial imagery, was added to the ArcGIS project file. If Bing Maps Aerial did not show a turbine installation, additional USGS orthoimagery, USDA/NAIP County Mosaic Orthoimagery, or Digital Globe imagery was added. For a data point where the turbine was not seen on an image, the conf_loc was entered as 0. If the turbine point showed partial construction or if other aspects were in doubt, the conf_loc was entered as 1. If an installed turbine was clearly seen on an image the conf_loc was entered as 2.

In addition to verifying the location of turbines already in the dataset, we also searched for new turbines. Visual aerial imagery searches were performed near known turbines and near facility locations found in the EIA and WENDI data. Any newly found turbines were digitized using a map scale of 1:2,000 to 1:3,000, then enlarged to a map scale of 1:500 to 1:1,000 for point adjustment. When complete, the state, county, image_name, image_year, and type_tower (see Data Records for description) were attributed to each turbine. Groups of turbines were then attributed to a facility by comparing the following characteristics across the EIA, WENDI, and DOF datasets: facility names, turbine counts, proximity to facility locations, and installation dates provided or acquired from comparing imagery through time. In addition, information found in publications such as press releases, industry websites, and photographs of named facilities were also used to verify the information found in the datasets. When possible, turbines were grouped and attributed to a common wind farm (site_name). In some cases, particularly with older turbines or locations where individual facilities overlapped, it was not possible to attribute individual turbines to distinct facilities. In these cases, a simple naming convention of the general geographic location accepted within the community of wind energy practitioners, or a county name and a numerical sequence beginning at 1 was used, for example, unknown County 1, unknown County 2, or unknown Facility Area 1.

To develop turbine specifications, the facility names (site_name) were used in web searches of additional industry publications and press releases to identify the manufacturer, model, and technical specifications of turbines installed at each facility. When turbine specifications were not reported, but make and model of the turbine were, we used turbine specification information provided by manufacturers for their turbine models. If no information was found or major discrepancies were found across sources of information, a confidence of 0 was entered under the conf_attr field. If some discrepancies were found or some uncertainty existed (usually described in comments), a confidence of 1 was entered. If the identification was apparent and easily confirmed across different sources of information, a confidence of 2 was entered.

After each state shapefile was completed, it was peer reviewed and corrected if necessary. Then, state shapefiles were merged into a single national file that was then peer reviewed by USGS analysts outside the development team. The first dataset was built using DOF data through September 2013, and made available to the public on February 11, 2014. After this, public comments that included new turbine locations and updated DOF turbine data were added to March 2014. In summary, of the 48,976 turbines, 34,885 have an ORS number and are part of the FAA DOF data. The remaining 14,091 were hand digitized and not part of the March 2014 DOF. Many of these are older, smaller turbines less than 200 ft in total height, and thus not reported to the FAA.

## Data Records

The data are distributed as public domain and available in multiple formats, including a web application and web service endpoints. A static copy of the data exists at the Data Dryad Repository (Data Citation 1). In addition, a static copy and potential future updates are available for download as USGS data series 817 from the USGS online publications directory (Data Citation 2).

Other data formats (KMZ, csv) and metadata are available (http://energy.usgs.gov/OtherEnergy/WindEnergy.aspx#4112358-data) then select the data tab. A web-based interactive map is also available (http://eerscmap.usgs.gov/windfarm/) which allows users to see and use an abridged version of the data through a web browser. Here the user can easily find a specified location and filter the dataset by total height, capacity, or blade length. The web application also contains a comment box, which has been used to report any overlooked single turbines not often listed in larger facility databases. In addition the following web service endpoints are available:

Tiled: http://eerscmap.usgs.gov/arcgis/rest/services/wind/wTurbinesWM/MapServer

Dynamic: http://eerscmap.usgs.gov/arcgis/rest/services/wind/wTurbinesWMDyn/MapServer

The dataset contains the fields detailed below. When a numeric data type value was not known, −99999 was entered to represent a null value and unknown was used for string data types.

**FID:** Object identification number. Automatically generated ID by ArcGIS.

**Shape:** Data type of features. Automatically generated by ArcGIS.

**unique_id:** Unique, stable object number for cross-reference, assigned by the USGS.

s**ite_name:** Name of wind energy facility associated with each turbine point. Can be either:

- Conventional industry name—whichever name is common between WENDI, EIA, and public information from web searches. Phases of development are denoted by numbers following the name, such as Bear Wind Farm 2.
- unknown County, unknown County # or unknown General Name was used when we could not determine a facility name from any public data, or where individual facilities overlapped and it was not possible to attribute individual turbines to distinct facilities.

**total_turb:** The number of turbines at a facility. This was calculated as the total number of turbines with the same unique site_name.

**on_year:** The year the turbine began producing power, if known. Can be either:

- The year the facility began generating, based on information from press releases and facilities shapefiles; multiple years indicate the project spanned more than one year, contained multiple phases of development, or began producing energy (went ‘online’) at some point within the first and last year listed; formatted as (year1)_(year2)_(year3).
- unknown: We could not determine the year.

**year_range:** Whether the on_year is a range (‘yes’) or a single year (‘no’). If on_year was ‘unknown’ year_range was ‘no’.

**on_year_s:** Earliest possible year the facility began generating, based on information from press releases and facilities shapefiles; the earliest year of on_year.

**manufac:** The name of the turbine manufacturer. Vesta, Siemens, Suzlon, etc., or ‘unknown’ if not determined.

**model:** The manufacturer's model name of each turbine, for example 1.5SLE, V100_1.8, Z50, etc., or ‘unknown’ if not determined.

**type_tower:** Description of the structural characteristics of the turbine tower:

- monopole—a single tubular column structure with nacelle and blades.
- pad only—no tower was observed but a pad was present.
- small generator—a building-mounted, small wind energy generator.
- small monopole—a monopole turbine less than 30.5 m tall as visually identified in imagery, or reported in DOF dataset (in some cases turbines below the reporting requirement of 200 feet, were included in the DOF dataset).
- trestle—a lattice type tower structure with nacelle and blades.
- small trestle—a trestle turbine less than 30.5 m tall as visually identified in imagery, or reported in DOF dataset.
- unknown—unidentified, unseen on imagery, or unknown turbine specifications.

**decommiss:** Whether the turbine was decommissioned:

- no—turbine is not known to be decommissioned, appears to be running, and publications confirm the same.
- yes—includes both image-verified removed turbines and ones found to be removed or no longer in service based on publications.

**MW_turbine:** Power generation capacity of the turbine in megawatts based on turbine model specifications or other sources.

**total_cpcy:** Total nameplate capacity in megawatts of the facility calculated as the sum of MW_turbine for records with the same site_name.

**total_ht:** Height of entire wind turbine from ground to tip of a vertically extended blade above the tower based on model specifications, in meters.

**tower_h:** Height of the tower only, based on model specifications, in meters.

**blade_l:** Length of the blade from model specifications when provided or one half of the diameter when not provided, in meters.

**rotor_dia**: Rotor diameter based on model specifications, in m.

**rotor_s_a:** Rotor swept area in m^2^: Pi*r squared, calculated as 3.14159 (([**rotor_dia**] /2)*([**rotor_dia**] /2).

**lat_DD:** The latitude of the turbine point in the dataset, in decimal degrees, using the projected coordinate system (PCS): North America Albers Equal Area Conic coordinate system.

**long_DD:** The longitude of the turbine point, in decimal degrees, using PCS: North America Albers Equal Area Conic coordinate system.

**state:** State in which the turbine is located using the 2-letter standard state postal abbreviation.

**county:** County where the turbine is located, no abbreviations.

**conf_attr:** The confidence in turbine attributes:

- 0—no confidence, no facility data, no name, nothing in publications.
- 1—partial confidence, incomplete information or discrepancies across data sources or other discrepancies found, usually explained in comments field.
- 2—full confidence: consistent information across multiple publicly available data sources.

**conf_loc:** Confidence in turbine location:

- 0—no confidence: nothing on image, image has clouds, never built, previously removed, or tower location needs newer imagery; for example, turbine built in 2013 but latest available imagery is 2011.
- 1—partial confidence: image shows a developed pad with concrete base and/or turbine parts on the ground.
- 2—full confidence: image shows an installed turbine or a tower being constructed, the tower is at least partially installed, crane and other equipment may be present.

**WENDI:** Facility name based on WENDI data, now available at openei.org.

**EIA:** Facility name based on EIA derived from 2012 EIA-860 data.

**FAA_jdate:** FAA Julian date, from FAA DOF date of notification or permit. USGS removed alpha characters from the original FAA string. Formatted as a 4-digit year followed by the day.

**FAA_AGL:** FAA height of turbine tower and extent of rotor, from FAA DOF. AGL=Above Ground Level elevation. USGS converted from feet to meters by dividing feet by 3.28.

**FAA_ORS:** FAA unique identifier for each turbine from FAA DOF. ORS=Obstacle Repository System. The first two digits indicate a state, and the sequence of numbers uniquely identifies each turbine.

**image_name:** Source of imagery used for visual analysis. Can be:

- Bing Maps Aerial—Esri ArcGIS Base maps.
- Digital Globe—the USGS uses the Rapid Delivery of Online Geospatial-Intelligence (RDOG) from a National Geospatial Intelligence Agency contract under NextView License which is updated continually.
- NAIP—National Aerial Imagery Program, USDA Ortho Imagery, from http://datagateway.nrcs.usda.gov/.
- USGS EDC SDDS—Ortho Imagery from EROS Data Center server. Available through The National Map (http://nationalmap.gov/ortho.html).

**image_year:** The year of the image used to verify a turbine’s position, usually only included for National Aerial Imagery Program (NAIP). Year is unknown for records with Esri ArcGIS base maps and Digital Globe.

**comments:** Data developer (author) comments to aid in attribution or additional explanations.

## Technical Validation

**Figure 1:**
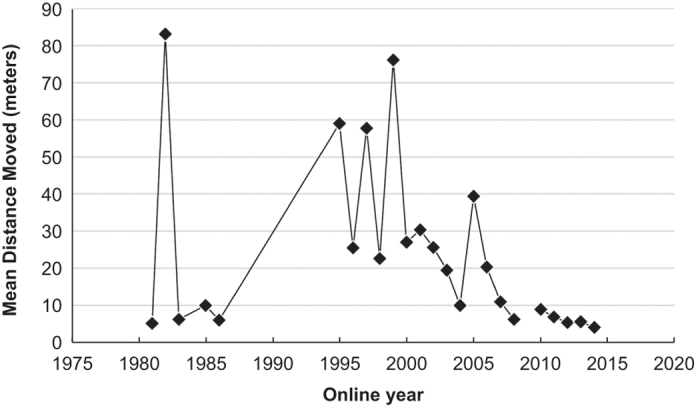
The mean distance, in meters, turbine points were moved when validating and correcting their locations by the first year a facility was operational (on_year_s in the dataset). Blanks in the time series represent years with no turbines.

Data were validated or quality controlled in a number of ways. First, the use of high-resolution aerial imagery and post-digitizing review of the locations by a different analyst both improved the accuracy of, and validated, turbine locations. We had multiple instances of shifts in sets of turbines likely caused by differences in the geodetic datum reported to FAA versus used when mapping or during geodetic datum transformations. Overall, the average distance we moved turbine points decreased through time, indicating newer turbines are more accurately mapped when self-reported in the FAA DOF (Figure 1).

In rare cases we found tall structures labelled as turbines that were not, and we also digitized 14,091 small turbines, that were not tall enough to be reported in the FAA DOF. These were mostly in southern California, where wind turbines were installed from the 1980s to present.

During the digitizing process we compared wind turbine locations digitized with Bing Maps Aerial base maps to those digitized using NAIP imagery and found them to be as accurate as, or better than, NAIP. NAIP provides 1-meter ground sample distance orthoimagery to within plus or minus 6 m to true ground at a 95-percent confidence level. In some locations the aerial imagery may have registration errors and internal inaccuracies. With these issues, and 4 m of additional human error associated with placing the turbine point at the base of the turbine tower (estimated when analysts checked each other’s work), the position of the data points is within 10 m. A small percentage of turbines in the dataset are not spatially validated as noted with a confidence in location of 0. This is because high-resolution imagery was insufficient, due to clouds obscuring the image or simply a lack of imagery for the area in question.

We strived to reduce human error by having analysts check each other’s work and by internal checks for data accuracy. When a state was complete, a different analyst reviewed all aspects of the data. This included checking turbine locations against aerial imagery and moving points that were misplaced or missed, checking turbine specifications against known specifications of specific turbine makes and models, and reviewing the information used to develop the turbine specifications and the final confidence rankings associated with each turbine. Once all the states were merged into a single file, we checked each attribute field for internal accuracy. For example, analysts sometimes used slightly different text entries for turbine makes such as GE 1.5 SL, GE1.5SL, or GE-1.5-SL. These types of text-formatting errors were found and corrected. We also calculated the total height of a turbine using attribute fields for hub height and blade length, then compared this to the FAA’s Above Ground Level field. When these values were different by more than 2 m, we rechecked the turbine specifications and their sources and made changes where necessary.

Finally the data and metadata were reviewed by individuals outside the development team. The USGS internal review process included feedback from 2 technical specialists and a metadata expert. In addition, the original data were made publicly available on February 11, 2014, with a web application that allowed users of the website to provide feedback. Since then, we received 20 comments that noted additions or corrections to data. We received a small number of comments regarding facility names or the makes and models of turbines at a facility. These comments were validated and then corrected to the database in the latest revision (March 2014).

**Figure 2:**
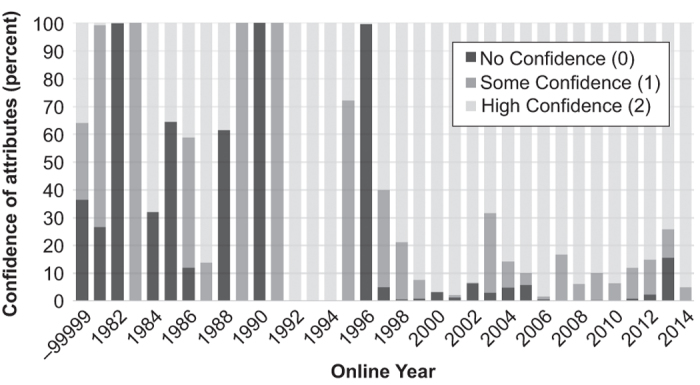
Stacked chart of the level of confidence in turbine attributes (the conf_attr field) by year. −99999 are turbines with unknown installation dates. These are typically older turbines.

Currently, of the 48,976 turbines, 69% (33,676) have an attribute confidence level of 2 (full confidence), 18% (8,772) have confidence of 1, and 13% (6,528) have confidence of zero. Levels of attribute confidence were much higher for more recent installations though we found a delay between the installation of facilities and when information about them becomes available, as shown by the slight decline in confidence in 2014 (Figure 2). For location confidence, 98% (48,081) of the turbines had a high value, 1% (656) medium, and less than 1% (239) had no confidence.

We note that a full validation of these data would require detailed information from each wind facility and a field campaign to map the base of each turbine. Doing so would be labor intensive, and in some cases wind producers may not want to provide facility level information. We emphasize that though we made many efforts to validate locations and enhance accuracy, none of turbines were field verified. Instead, the dataset ultimately represents a collection, synthesis and crosschecking of publicly available data, which was found from internet searches and aerial imagery.

However, a dataset did exist that allowed a level of validation. Another scientist (T. Katzner) developed an unpublished dataset of 11,181 turbines in California while studying raptor interactions with wind farms. We compared our turbine data to his for the same geographic area. Of the 11,181 turbines in the Katzner data, 46 were not in the original USGS dataset. Of these 46, 38 were considered questionable (the imagery did not confirm a turbine at the location). Of the remaining 8, 2 were confirmed misses, and the remaining 6 were small, residential turbines. For the same area of California, the USGS dataset contained 14,524 turbines. Of the 3,343 not in the Katzner data, 340 came from the Sky River wind facility (including 5 decommissioned), 2,709 were mapped as decommissioned, and the remaining 294 were present in the USGS data but not in the Katzner data.

## Usage Notes

A main caution associated with using the dataset is the −99999 used as the null data value. Shapefiles do not support null values, and we used −99999 to represent this. Before performing any mathematical operations on numerical fields (averaging tower height for example), the −99999 values should be converted to true nulls.

In addition, the dataset should not be used as a source for comprehensive information on small turbines installed on private property. These types of turbines are nearly impossible to find in imagery and no repository exists for information on these small turbines. Those that exist in the dataset were reported to us or found by chance. We did not make a systematic effort to map these non-industrial turbines.

As shown in Figure 2, the confidence for turbine specifications is generally low for older facilities. We simply could not find information about these older facilities and the makes and models of turbines installed in them. Thus, caution is warranted for any application that uses these particular data. Turbine specifications for newer facilities are relatively well established and can be used with more confidence.

Finally, while we included decommissioned turbines, the data are a not a comprehensive history of all decommissioned turbine locations. Only those turbines entered as decommissioned in the FAA DOF, or where we found evidence of decommissioning, exist in the data. Many other turbines may have been decommissioned and either not reported to the FAA or missed by our mapping efforts. For example, smaller turbines, such as the multiple thousands installed, torn down, and replaced in southern California have never been mapped to our knowledge and no data exist to reconstruct this history.

## Additional Information

**How to cite this article:** Diffendorfer, J. E. *et al.* Onshore industrial wind turbine locations for the United States up to March 2014. *Sci. Data* 2:150060 doi:10.1038/sdata.2015.60 (2015).

## Supplementary Material
